# Genome-Wide Mutational Signature of the Chemotherapeutic Agent Mitomycin C in *Caenorhabditis elegans*

**DOI:** 10.1534/g3.115.021915

**Published:** 2015-11-11

**Authors:** Annie S. Tam, Jeffrey S.C. Chu, Ann M. Rose

**Affiliations:** * Department of Medical Genetics, University of British Columbia, Vancouver, V6T 1Z3, Canada; † Terry Fox Laboratory, British Columbia Cancer Agency, Vancouver, V5Z 1L3, Canada; ‡ WuHan Frasergen Bioinformatics Co. Ltd., Wuhan, Hubei Province, 430075, China

**Keywords:** whole genome sequencing, *C. elegans*, model organism, mutation spectrum, mitomycin C

## Abstract

Cancer therapy largely depends on chemotherapeutic agents that generate DNA lesions. However, our understanding of the nature of the resulting lesions as well as the mutational profiles of these chemotherapeutic agents is limited. Among these lesions, DNA interstrand crosslinks are among the more toxic types of DNA damage. Here, we have characterized the mutational spectrum of the commonly used DNA interstrand crosslinking agent mitomycin C (MMC). Using a combination of genetic mapping, whole genome sequencing, and genomic analysis, we have identified and confirmed several genomic lesions linked to MMC-induced DNA damage in *Caenorhabditis elegans*. Our data indicate that MMC predominantly causes deletions, with a 5′-CpG-3′ sequence context prevalent in the deleted regions of DNA. Furthermore, we identified microhomology flanking the deletion junctions, indicative of DNA repair via nonhomologous end joining. Based on these results, we propose a general repair mechanism that is likely to be involved in the biological response to this highly toxic agent. In conclusion, the systematic study we have described provides insight into potential sequence specificity of MMC with DNA.

Mitomycin C (MMC) is a toxic antitumor antibiotic that is clinically used to treat many types of cancers (Kato *et al.* 1979; Spain 1993; Michalaki *et al.* 2010; Greenberg *et al.* 1982; Doll *et al.* 1985). The antitumor activity of MMC has been attributed to its potent DNA crosslinking ability, which strongly inhibits DNA replication and transcription (Iyer and Szybalski 1964; Tomasz *et al.* 1974). The chemical mechanism of MMC has been well documented *in vitro*: MMC preferentially interacts with guanines, the reactivity of which is affected by the sequence context, and additional studies have implicated specificity of interstrand crosslink formation at 5′-CpG-3′ sequences (Gargiulo *et al.* 1995; Kumar *et al.* 1992; Borowy-Borowski *et al.* 1990). Early studies in different organisms identified many biological consequences of MMC treatment that include changes in recombination pattern, chromosome interchanges, sister-chromatid exchanges, tandem-base substitutions, and deletions (Schewe *et al.* 1971a,b; Natarajan *et al.* 1983; Russo *et al.* 1993; Takeiri *et al.* 2003). The *in vivo* data indicate that a wide range of genetic lesions can be caused by MMC. However, the biological frequency, as well as the different types of mutations found across the entire genome have never been studied with high resolution methods . To understand the biological consequences of clinically used mutagens, it is imperative to first characterize the spectrum of mutagenic changes caused by these agents in a biological context.

The nematode *Caenorhabditis elegans* is a well characterized genetic model system with which to study genomic damage generated by exposure to chemical agents. Recent studies have utilized this model organism to identify whole genome mutational profiles of a multitude of genotoxic agents (Flibotte *et al.* 2010; Sarin *et al.* 2010; Meier *et al.* 2014). *C. elegans* presents an excellent genetic model due to the ease of capturing and maintaining specific mutations using specialized chromosomes known as genetic balancers (Jones *et al.* 2011). A class of genetic balancer, the reciprocal translocation, can be used to stably maintain a specific lethal mutation disrupting an essential gene by preventing recombination between the wild-type homolog and mutation-bearing chromosome. It is clear from the published literature that MMC causes mutations that can be very large and complex, which may be lost and therefore not recovered if an essential gene is disrupted. Therefore, we used the genetic balancer *hT2* to capture and maintain lethal mutations caused by MMC, first as a proxy of damage for the rest of the genome, and also as a way to capture types of mutations which may be lost by inactivation of an essential gene in nonbalanced chromosomes. To evaluate our method for optimal detection of variants, we manually characterized lethal mutations generated in our screen and tested the bioinformatics software for the ability to detect these same mutations in an unbiased fashion.

In our approach, we used a two-part, complementary method to identify variants caused by MMC treatment in *C. elegans*. The first method involved generating and genetically mapping recessive lethal mutations with the genetic balancer *hT2* to capture the state of the genome immediately after mutagenesis. The chromosomes balanced by *hT2* were maintained in a heterozygous state, and therefore represent a catalog of the extent of damage caused by MMC. The second method involved extension of this analysis to the rest of the genome, cataloguing mutations that were maintained in a homozygous state. Genome-wide analysis directly provided information about the frequency of mutations induced by MMC. The methods employed in our analysis allowed us to understand the consequences of MMC treatment in a biological system.

## Materials and Methods

### *C. elegans* strains and culture conditions

Wild-type and mutant *C. elegans* strains were cultured in Petri dishes on agar nematode growth medium (NGM) streaked with *Escherichia coli* OP50 (Brenner 1974). *C. elegans* were maintained at 20° as previously described (Brenner 1974). The nomenclature for genes and alleles follows the uniform system adopted for *C. elegans* (Horvitz *et al.* 1979). Strains were obtained from the Caenorhabditis Genetics Center (CGC) unless otherwise indicated. The genetic balancer *hT2(I;III)* was induced by gamma irradiation (McKim *et al.* 1993), and inserted with a transgene that expresses the dominant pharyngeal GFP marker *Pmyo-2*::*GFP*. The region balanced by *hT2(I;III)* has previously been identified to span the left of chromosome I, and the right of chromosome III (McKim *et al.* 1988, 1993). All mutations denoted with the *h* prefix originated from the Rose laboratory.

### Mutagenesis and forward genetic screen

An optimal 750 µM dose of MMC, based on findings in *Drosophila melanogaster*, was used to treat KR4949 nematodes of genotype *hT2[bli-4(e937)] let-? (q782) qIs48]* I; III /*unc-13 (e51)*, *dpy-5(e61*) (Schewe *et al.* 1971a,b). The mutagen was prepared by dissolving 2 mg of MMC in 200 µl dH_2_O, followed by dilution with M9 buffer to give final concentrations. Two vials of MMC were used to account for batch variability. Concentrations at 375 µM, optimal dose (750 µM), and 1400 µM were used to determine differences in potency. The P_0_ hermaphrodites, subsequently referred to as *dpy-5unc-13 / hT2* were washed off plates with M9 buffer, collected by centrifugation, and soaked with MMC for 4 hr at 20°. After 3 d, the F_1_ progeny were screened for the absence of Dpy-Uncs indicating lethal mutations captured in the *hT2*-balanced region. Single F_1_ animals from the P_0_ hermaphrodite plates were placed onto individual Petri plates, and the F_2_ animals were screened for recessive lethal mutations. Due to linkage with the visible markers, the absence of mature Dpy-Unc animals either indicated the presence of a lethal mutation in the balanced region of chromosome I or that a lethal mutation was in the balanced region of chromosome III due to pseudolinkage. Isolated lethal mutation-bearing strains were frozen.

### Genetic mapping

Three-factor mapping was used to determine the genetic location of the lethal mutations. The *dpy-5unc-13 / hT2* lethal mutation-bearing strains were crossed to N2 males. Non-GFP L4 F_1_ hermaphrodites were picked from each strain and transferred daily over 4 d. Each brood was scored for wild-type, Dpy-Unc, Dpy, and Unc animals. Genetic distance of the putative lethal mutations were calculated using the equation for recombination frequency (*p*) = $1-\sqrt{1-2R}$(Brenner 1974), where R denotes the fraction of 2 x Dpy-Uncs (recombinants) over 4/3 wild-type (to calculate total progeny).

### Genomic DNA extraction for whole genome sequencing

Ten lethal mutation-bearing strains KR4968, KR4969, KR4978, KR4984, KR4995, KR5006, KR5009, KR5034, KR5035, and KR5037 were prepared for whole genome sequencing. These strains carried the lethal mutations *h2717*, *h2718*, *h2727*, *h2733*, *h2744*, *h2755*, *h2758*, *h2784*, *h2785*, and *h2787* on chromosome I. The genomic DNA preparation pipeline has previously been described (Chu *et al.* 2012). The Illumina HiSequation 2000 sequencing platform was used for whole genome sequencing at the Michael Smith Genome Sciences Centre, Vancouver, B.C., Canada. Clusters were generated on the Illumina cluster station and paired-end reads were generated following the manufacturer’s instructions. The V1.0 Illumina Genome Analyzer analysis pipeline was used for image analysis, base calling, and error calibration.

### Data processing and bioinformatics analysis

Genomic sequences were aligned to the annotated sequence of *C. elegans* available on WormBase (WS200) (http://www.wormbase.org) using BWA at the default setting (Li and Durbin 2009). A composite parental strain was derived from combining the bam files of two strains used to make the parental strain. Integrative Genomics Viewer (IGV) was used to visualize and browse genomes (Robinson *et al.* 2011). Single nucleotide variants (SNVs) were called using VarScan with the following parameters: –min-coverage 3 –min-avg-qual 5 –*P*-value 0.1 –str-filter 0 –min-freq-homozy 0.9 (Koboldt *et al.* 2009). To identify strain-specific events, each sequenced strain was compared to the other nine strains, as well as a composite parental strain. A custom script was used to parse each strain for SNVs that were present in one strain and absent in the parental and other nine strains. The identified SNVs were then pooled for analysis. The distribution and number of SNVs were compared to 10 spontaneously mutating N2 strains and 10 EMS-treated strains using raw data from Denver *et al.* (2009) and Flibotte *et al.* (2010), respectively. *P*-values of the distribution of SNVs were derived from chi-square statistics applied to the distribution of SNVs (*P* < 0.05). Insertions and deletions (indels) were called using Pindel with default parameters, and set to filter indels occurring in homopolymer regions (Ye *et al.* 2009). A custom script was used to parse each strain for homozygous indels that were unique to each strain and absent in the other sequenced strains and composite parental. These strain-specific indels were independently verified on IGV and the left and right 15 flanking nucleotides (greater than one helical turn of DNA) were extracted to provide regional context of indels. A custom script was used to parse and tally the number of dinucleotide combinations in the deleted segments of DNA, left and right 15 flanking nucleotides of the deletions, and chromosome I of an untreated N2 strain. The *hT2* breakpoint was determined by arranging segments of chromosome I and III into 5 kb or 10 kb bins, then major drops in average coverage were identified with a custom script at the previously mapped genetic locations (McKim *et al.* 1988, 1993). The breakpoints were then manually identified using IGV by matching misaligned reads.

### Physical identification of putative lethal mutation sites

Confidence limits of 95%, calculated using binomial distribution statistics applied to the map units, were used to determine the genetic map range. This range was used to estimate the physical location of each mutation, and the locations of the closest known genes were used as starting and end points of analysis. The putative lethal mutation sites were identified in the mapped regions using manual curation with IGV scanning the entire mapped range. The identified lethal mutations were additionally used to test the sensitivity of Pindel. The putative lethal mutation sites were identified with the following criteria: (1) allelic ratio was approximately 50%, indicating heterozygosity and therefore the presence of the genetic balancer; (2) the identified mutations fell within the range determined by three-factor mapping; (3) the mutation was unique to the strain analyzed.

### Data availability

Strains are available upon request. The whole genome sequencing data has been deposited in the National Center for Biotechnology Information Sequence Read Archive under accession number PRJNA294365.

## Results

### Forward mutation frequency of mitomycin C-induced recessive lethal mutations

The forward mutation frequency in *C. elegans* is a measure of lethal events recovered using a genetic balancer. Specifically, in this study, the frequency was calculated as the number of recovered lethal mutations divided by the total number of F_1_ animals screened. This number gives an estimate of the potency of a specific mutagen and in this study also served as a baseline of mutagenesis. The forward mutation frequency of MMC was calculated to range from 2.2 to 5.2%, approximately two to five lethal mutations per 100 haploid copies of the region balanced by *hT2* (Table 1). This covered approximately one-fifth of the genome (18.2 Mbp). Differences in forward mutation frequency were observed between the two different batches of MMC and not between the optimal dose and half the optimal dose (375 µM). The low forward mutation frequency at 1400 µM was most likely due to induced sterility of the P_0_ animals, indicated by the low number of lethal mutations recovered in the screen. The forward mutation frequency for a number of mutagens has been determined in *C. elegans* using the reciprocal translocation *eT1* to capture lethal mutations (Rosenbluth and Baillie 1981; Adames *et al.* 1998; McKim *et al.* 1988, 1993; Johnsen and Baillie 1988; Rosenbluth *et al.* 1985; Stewart *et al.* 1991). Using *eT1*, the forward mutation frequency for MMC was observed to be 2% with an equal number of lethal mutations recovered in the first and second broods (A. M. Rose, unpublished results). This compares favorably with gamma irradiation (4.0%) (Rosenbluth *et al.* 1985), UV-TMP (3.3%, A. M. Rose, unpublished results), UV irradiation [3.1% (Stewart *et al.* 1991)], and formaldehyde [1.6% (Johnsen and Baillie 1988; Moerman and Baillie 1981)], but using this measurement, MMC does not appear to be as potent at inducing lethal mutations as the monoalkylating agent EMS [10.3% (Rosenbluth 1983)].

**Table 1:** Forward mutation frequencies of recessive lethal mutations induced by mitomycin C

Batch	Dose (µM)	Number of F_1_s Tested	Number of Lethal Mutations	Forward Mutation Frequency (%)*^a^*
1	750	437	24	5.5
1	750	550	25	4.5
1	750	436	24	5.5
2	375	145	3	2.1
2	750	580	13	2.2
2	1400	145	1	0.7

a Forward mutation frequency was calculated as a percentage of lethal mutations – (isolated lethal mutations/F1s tested) × 100%. Refer to text for details.

### Genetic mapping of mitomycin C-induced lethal mutations

The forward genetic screen produced 90 MMC-induced lethal mutation-bearing strains. Of these 90, 14 did not survive the freezing process and were not further analyzed. The lethal mutation was lost from 7 strains prior to mapping, identified by the loss of the GFP marker, which indicated balancer breakdown. Using three-factor mapping, 69 strains carrying MMC-induced lethal mutations were mapped to chromosome I or III (Supporting Information, Table S1). 21 lethal mutations were mapped to chromosome I and 48 lethal mutations were mapped to chromosome III. The presence of the visible markers *dpy-5* and *unc-13* on chromosome I allowed for recombination mapping of the lethal mutations relative to the visible markers on this chromosome. Of these lethal mutations, 10 (47.6%) were to the left of *dpy-5*, 10 (47.6%) were to the right of *unc-13*, and 1 was positioned between *dpy-5* and *unc-13* (Figure S1). The map distance for each of the mutations was determined by calculating the 95% confidence intervals as described in the *Materials and Methods*.

### Identifying mitomycin C-induced lethal mutations using three-factor mapping data

Ten of the 21 chromosome I lethal mutation-bearing strains were sent to the Michael Smith Genome Sciences Centre for whole genome sequencing. These strains carried the lethal mutations *h2717*, *h2718*, *h2727*, *h2733*, *h2744*, *h2755*, *h2758*, *h2784*, *h2785*, and *h2787*. The genome sequences were first analyzed for the presence of the balancer, indicated by heterozygosity of the *dpy-5* and *unc-13* markers, as determined by the ratio of reference:variant reads visualized using IGV (Table S2) (Robinson *et al.* 2011). Using three-factor mapping data and manual curation with IGV, putative lethal mutations were identified for 7 of the sequenced strains carrying *h2717*, *h2718*, *h2727*, *h2733*, *h2755*, *h2758*, and *h2787* (Table 2). Manual curation with IGV was not able to identify the lethal mutations *h2744*, *h2784*, and *h2785*. Analysis included scanning the entire mapped range for single nucleotide changes, indels, deletions, and insertions. However, it is possible that the strains carrying these lethal mutations harbor other complex variations. All of the identified putative-lethal mutations were deletions that ranged from 8 bp to 318,826 bp distributed along the *hT2*-balanced chromosome I region that spans approximately 11 Mbp (Figure S1). The boundary of *hT2* rearrangement was identified by segmenting chromosome I into 5 kb or 10 kb bins and examined for changes in average coverage at the estimated genetic positions (McKim *et al.* 1988, 1993). We found the exact breakpoints of *hT2* by examining in detail the sequencing reads with soft-clipped sequences and abnormal mapping pairs and found *hT2* had resulted from a series of complex rearrangements at chromosome I:13187121 and chromosome III:4989662 (Figure S2).

**Table 2:** Mitomycin C predominantly induced deletions of varying sizes on chromosome I

Strain	Allele	Identified Genetic Mutation	Mapped Location (Map Units)	Physical Location (bp)	Number of Genes Potentially Affected	Predicted Essential Gene
KR4968	*h2717*	318,826 bp deletion	−13.23 ± 2.0	I:753,745-1,072,570	56	N/A
KR4978	*h2727*	2382 bp deletion	−12.63 ± 1.5	I:1,742,835-1,745,216	1	*mppa-1*
KR4984	*h2733*	8 bp deletion	−11.62 ± 2.7	I:1,833,730-1,833,737	1	*C53H9.2*
KR5009	*h2758*	4962 bp deletion	−12.10 ± 2.1	I:2,100,906-2,105,867	1	*npp-13*
KR4969	*h2718*	21 bp deletion	−6.09 ± 2.0	I:2,897,024-2,897,046	1	*Y71F9AL.17*
KR5037	*h2787*	15,059 bp deletion	−2.06 ± 1.6	I:5,112,626-5,127,686	4	*mat-1*
KR5006	*h2755*	4877 bp deletion	Between markers *dpy-5* (0.00 ± 0.002) and *unc-13* (2.07 ± 0.004)	I:6,175,541-6,180,417	1	*T09B4.9*

### Insertion and deletion analysis using Pindel

The identification of the lethal mutations provided an indication of the types of mutations that might be induced genome-wide. Analysis was extended to the rest of the genome by using Pindel to identify deletions in the 10 sequenced genomes (Ye *et al.* 2009). A custom script comparing the read support of a deletion to the average depth across a deletion was used to parse the Pindel data for strain-specific events. This analysis revealed 22 strain-specific deletions that were in the 10 strains, all of which were manually examined using IGV (Table S3). Consistent with the analysis of the balanced regions of MMC-treated animals, MMC also induced deletions of varying sizes in the unbalanced genome. The deletions identified using Pindel ranged from 2 bp to 13,671 bp (Table 3). The global Pindel deletion analysis was able to accurately identify the seven heterozygous lethal mutations that were identified manually using IGV, confirming the ability of Pindel to robustly call variants. Using these parameters, Pindel was also used to analyze the sequencing data for insertions in the 10 sequenced genomes. Four insertions in 10 strains were identified and verified by manual curation on IGV, and did not reveal sequence specificity. Insertions were not identified with manual curation of lethal mutations in the balanced regions of DNA, and very few were identified in the unbalanced regions. The few insertions observed in the unbalanced regions of DNA may have spontaneously occurred, since no insertions were identified in the chromosome I balanced region of DNA.

**Table 3:** Genome-wide sequence context of mitomycin C-induced deletions identified by Pindel

Origin of Deletion	Method of Initial Detection	Deletion Size (bp)	Sequence*^a^*
*h2727*	Pindel	2	TTCTTTTCATTTCTCtaTGTTTGCCTATCACT
*h2758*	Pindel	4	TAATTTCACTGGAAtcatTGTCTTCCTTATCAC
*h2718*	Pindel	4	ATCCAATTTTCCGC**cgcg**CAGTCGCCAAAAAGG
*h2755*	Pindel	5	TAAATGACTACTgtaa**cG**CTTGTGTCGATTTA
*h2718*	Pindel	5	TTCTAGGCATATattg**cG**AAATATCTTTATAA
*h2718*	Pindel	5	CTTGCGTGGGACCAgt**cg**tGTGGTCGAAACAGAC
*h2718*	Pindel	5	CAAATTGGAATGCTGc**cg**aaCTCTTGCCCATGTCT
*h2733*	Pindel	6	TTTGGAGCTGTCGAca**cg**ttCCGCGCCGCACTATA
*h2733*	Pindel	7	CATAAATCGCAAActg**cg**ttCTTCAGCAACAATAT
*h2733*	IGV	8	ATTTTCTGGTGAGTGa**cgcg**ataAAATCAATATTTTCT
*h2787*	Pindel	8	ATATTCGTGAAGAcattgttcCAACGCTGCACAATT
*h2718*	Pindel	9	GTGACCTTAAGAAgaa**cg**agatGAGATGGAGAGTTGA
*h2787*	Pindel	10	TCCAACACAGAGAtgc**cg**tag**cg**TGCAAATTAGGCTTT
*h2718*	Pindel	13	ACTGCATCTGgaattc**cg**atat**cG**ATTTCACGATCGAA
*h2727*	Pindel	22	AAAacca**cg**cagg**cg**a**cg**cctacatACCACGCAGGCAGCC
*h2718*	IGV	21	GACtcctcagcttcagcagaatacTCCTCAGCTTCAGCAGAATATCCATG
*h2727*	Pindel	58	CTTTTATACGTAACCtttcc < 48 > ataaaTGAAAAATTGCTTCC
*h2733*	Pindel	77	GACGACGACGAGGAAccccc < 67 > aaagcACCACGTGGAGATTA
*h2744*	Pindel	104	TGTTTCAAAATATACatctg < 94 > ctaatATAAATATCCATGCC
*h2717*	Pindel	165	AGtgccaacaacaatgtattc < 137 > caacaatcaTGCCAACAACAATGC
*h2717*	Pindel	331	GAGGAAAAGGATAACacatt < 321 > aaaaaTCGCAAAAAACGCAT
*h2727*	Pindel	394	TGGTTCGGCCAATGAatttt < 384 > ctcagTGTTTACGGTTTATA
*h2733*	Pindel	1003	ATAGAGAATATACGGtatgc < 993 > gcaatTATCAGATTTCTTGT
*h2727*	IGV	2382	ACATTCCTTTTCCGGggc**cg**<2372 > tcattTTTACGCCTGTCAG
*h2755*	IGV	4877	CCTCATTTTGTGTGTtaatt < 4867 > ttaatTCACTATAATCCAAA
*h2758*	IGV	4962	AATATCTTCAAGCCGtcttc < 4952 > atcacCATTAAATTATTAGT
*h2755*	Pindel	13,671	ATCCCACTTTGTAGAagaac < 13661 > ggagtTCCAAAGAGTCATGC
*h2787*	IGV	15,059	GACAATTCGAGAGAAagaat < 15049 > ttgtgTTTGCGGAGTCGCAT
*h2717*	IGV	318,826	CGTGAGTCGTTCCGAttggc < 318816 > tt**cg**tAGATAAAACTATTA

a Sequence shown includes deleted segments (underlined lower case nucleotides), flanking nucleotides (upper case nucleotides), 5′-CpG-3′ dinucleotides where visible (bolded nucleotides), and microhomology flanking the deletion junctions (double underlined nucleotides). IGV, integrative genomics viewer.

### Sequence context of mitomycin C-induced DNA damage

**Figure 1:**
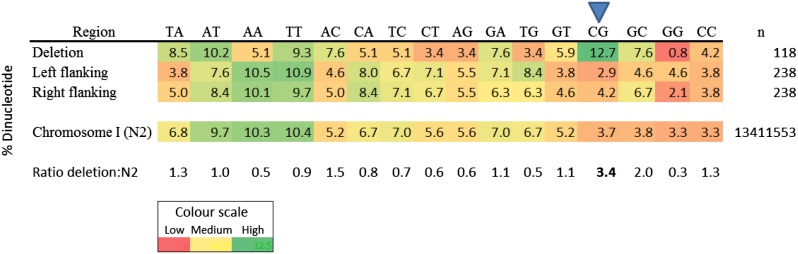
Putative mitomycin C (MMC)-induced deleted regions of DNA were enriched for 5′-CpG-3′ dinucleotides. Heatmap representation of the proportion of dinucleotide combinations in the putative MMC-induced deleted regions of DNA, 15 nucleotides flanking left and right, and an N2 *C. elegans* strain. Red represents a low number of observed events, while green represents a high number of events. n represents total number of nucleotides. Blue triangle indicates the 5′-CpG-3′ dinucleotides.

The sequence context of MMC-induced mutations was assessed by extracting the 15 flanking nucleotides around the deletions for analysis. The sequence context in the deleted regions of DNA revealed a prevalence of 5′-CpG-3′ dinucleotides *vs.* the other dinucleotide combinations (Figure 1). In addition, comparison of the deleted regions of DNA with the 15 nucleotides flanking both sides of the deletion revealed a higher proportion of 5′-CpG-3′ dinucleotides in the deleted regions of DNA *vs.* the flanking regions. On comparison with an N2 *C. elegans* strain, the 5′-CpG-3′ dinucleotide content in the deleted regions of DNA was 3.4-fold higher than in N2. This 5′-CpG-3′ dinucleotide footprint is consistent with *in vitro* data (Gargiulo *et al.* 1995; Kumar *et al.* 1992; Borowy-Borowski *et al.* 1990). Analysis of flanking regions around the deletion breakpoints revealed microhomology in 20 of the 29 deletions, ranging from 1 bp to 20 bp (Table 3). The methods used to characterize the spectrum of mutations in the balanced and unbalanced regions of DNA indicate that MMC interacts with preferred sequences in the DNA, leaving behind an identifiable footprint associated with deletions of variable size.

### Mitomycin C does not appear to induce single nucleotide variants genome-wide

**Figure 2:**
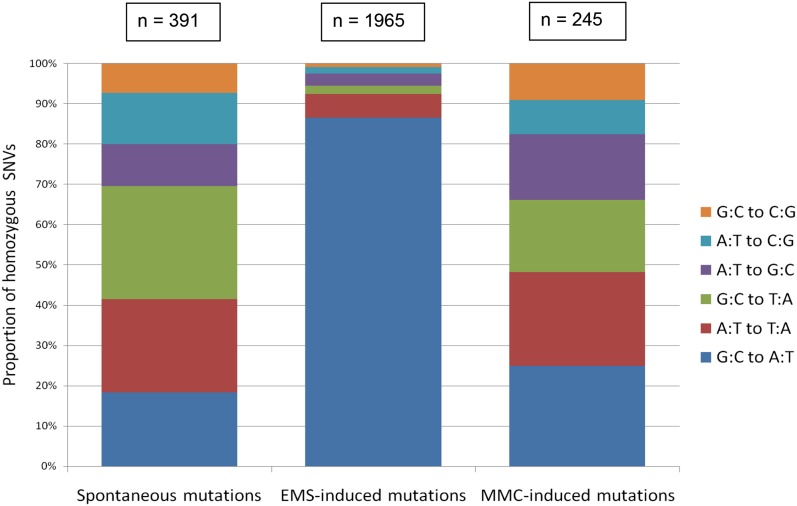
Genome-wide profile of mitomycin C (MMC)-induced single nucleotide variants (SNVs), compared to N2 and EMS-treated animals. Untreated N2, EMS-treated, and MMC-treated strains were compared to determine the distribution of homozygous SNVs in each strain relative to one other. The total number of SNVs in the MMC-treated animals (column 3) was similar to a spontaneously mutating strain (column 1), and much lower than EMS-treated strains (column 2). In addition, distribution of SNVs in the MMC-treated animals (column 3) was not significantly different from the untreated N2 strains (column 1), and the distribution of SNVs in the EMS-treated strains (column 2) differed dramatically from either spontaneously mutating or MMC-treated strains.

The 10 sequenced MMC-treated strains were analyzed for unique, homozygous SNVs using VarScan (Koboldt *et al.* 2009). SNVs were called by aligning to the *C. elegans* reference genome (WS200). Based on an approach described previously, homozygosity was defined as a reference to variant read ratio that was greater than 90% (Chu *et al.* 2012). For statistical purposes, only SNVs that had coverage greater than seven overlapping reads were included. SNVs that were present in the composite parental strain or occurred in more than one of the 10 sequenced MMC-treated strains were removed. VarScan and subsequent filtering identified 245 strain-specific, homozygous SNVs in the 10 strains. These SNVs were pooled for further analysis since each SNV represented a unique event. Variants were grouped as complementary base pairs – G:C > C:G, A:T > C:G, A:T > G:C, G:C > T:A, A:T > T:A, G:C > A:T – where the colons signify the complementary base pairs and the arrow (>) signifies the substitution. The distribution and number of SNVs were compared to 10 spontaneously mutating N2 strains (Denver *et al.* 2009) (Figure 2). The distribution of SNVs in the MMC-treated strains does not appear to be skewed toward any type of single nucleotide change, and was not statistically different from the distribution of SNVs in the untreated N2 strains. To further compare the frequency and distribution of SNVs, the 10 MMC-treated strains were also compared to 10 sequenced strains treated with EMS, a known monoalkylating agent with a distinct genetic profile (Figure 2) (Flibotte *et al.* 2010). The distribution of EMS-induced SNVs differed significantly from both spontaneously acquired and MMC-induced SNVs, as expected. Consistent with published results, the greatest proportion of SNVs in the EMS-treated strains were G:C > A:T events (Flibotte *et al.* 2010). The total number of SNVs identified in each strain is summarized in Table S4. Our analysis did not reveal many SNVs in the MMC-treated animals (245 strain specific SNVs in 10 strains). In fact, the SNVs identified in the MMC-treated strains were similar in number to a spontaneously mutating N2 strain (391 strain specific SNVs in 10 strains), and approximately eightfold lower than EMS-treated strains (1965 strain specific SNVs in 10 strains). MMC does not appear to induce many single nucleotide changes, as indicated by the similar number of events compared to spontaneously mutating strains. Analysis with IGV also revealed low number of SNVs, both homozygous and heterozygous, in balanced regions of DNA.

## Discussion

This study identified the genome-wide effects of treatment with MMC, a chemotherapeutic agent used in the clinic to treat a variety of cancers (Kato *et al.* 1979; Spain 1993; Michalaki *et al.* 2010; Greenberg *et al.* 1982; Doll *et al.* 1985). Using *C. elegans* as a model to simulate the genome in a noncancerous state in humans, we have identified a mutational profile that agrees with the reported chemistry of this drug (Tomasz 1995; Suresh Kumar *et al.* 1997). Using our methods to isolate mutational events, we have determined that MMC predominantly induces deletions, and does not appear to cause many single nucleotide changes and insertions (Table 2 and Figure 2).Our deletion analysis corroborates findings in previous studies, and we have additionally identified the frequency and distribution of these mutations on a genome-wide scale(Takeiri *et al.* 2003).To the best of our knowledge, this is the first high resolution study in which the genome-wide distribution of mutations caused by MMC has been reported in any organism.

### Mitomycin C is a potent mutagen with a clear genetic footprint

We defined the optimal dose in our study as one that induced the maximum number of lethal mutations without causing sterility. By testing three concentrations, our results indicate a lower concentration was sufficient to induce the same number of lethal mutations compared to the optimal dose (Table 1). In addition, by testing two batches of MMC to account for stability differences, we identified batch variability, which has previously been reported for this drug (Woo *et al.* 1997; Cervenka and Hirsch 1983). By comparing the forward mutation frequency for lethal events recovered using the balancer *hT2* with the published frequencies of known mutagens using the balancer *eT1*, the data revealed MMC to be a potent mutagen among these tested genotoxic agents (Rosenbluth and Baillie 1981; Adames *et al.* 1998; McKim *et al.* 1988, 1993; Johnsen and Baillie 1988; Rosenbluth *et al.* 1985; Stewart *et al.* 1991). Our analysis indicates that MMC predominantly causes deletions (Table 2 and Table 3), and does not appear to cause other mutations such as insertions or single nucleotide changes (Figure 2). Taken together, MMC is a potent mutagen that interacts with DNA in a specific manner that results in a predictable mutational background. Therefore, MMC could be a useful mutagen to include in *C. elegans* knockout protocols due to its specificity of inducing deletions with minimal background mutations (The *C. elegans* Gene Knockout Consortium). Should MMC be used in mutagenesis screens, our data indicate that a concentration of 375 µM is sufficient to induce lethal mutations in *C. elegans*, and that using the same vial of MMC will control for batch variability.

### Mitomycin C predominantly induces deletions in a sequence-specific context

We identified specificity of MMC in inducing small to large deletions, but did not find a statistically significant increase in single nucleotide changes and insertions (Table 2, Table 3, and Figure 2). Analysis of the deletions indicates a preference for a 5′-CpG-3′ sequence context, consistent with both *in vitro* and *in vivo* data (Figure 1 and Table 3) (Tomasz and Palom 1997; Tomasz *et al.* 1987, 1988; Palom *et al.* 2000; Takeiri *et al.* 2003). This sequence specificity has interesting consequences, as CpG islands overlap the promoter regions of 60–70% of all human genes (Illingworth and Bird 2009). In addition, the presence of CpG dinucleotides is elevated in exons and upstream regions of genes when compared to the CpG background level (Saxonov *et al.* 2006). The antitumor activity of MMC is thought to be due to blocks in DNA replication and transcription caused by the DNA-MMC crosslink (Iyer and Szybalski 1964; Tomasz *et al.* 1974). However, MMC-induced damage in CpG-rich promoter, exon, and gene upstream regions could alter gene expression, which might also contribute to cell death. By extracting the flanking nucleotides around the deletions for analysis, we also identified microhomology (1 bp to 20 bp) between the junctions of most of the deletions (Table 3). An earlier mouse study characterizing MMC-induced mutations captured with λ DNA-integrated chromosomes identified tandem-base substitutions, deletions with 2–6 bp or no microhomologies, and a lack of single nucleotide substitutions (Takeiri *et al.* 2003). Consistent with this study, our results also indicate that, unlike *in vitro* studies that report the induction of single nucleotide changes by MMC, the *in vivo* mutational spectrum does not fully reflect the types of DNA changes identified *in vitro*, possibly due to absent protein components in the *in vitro* systems (Srikanth *et al.* 1994). By identifying the mutational profile of MMC *in vivo*, we have assessed the genome-wide frequency of mutations generated by this chemical, and also contributed to an existing body of evidence that indicates model organism-based *in vivo* studies better capture the damage that could occur in human cells.

### Possible mechanism of mitomycin C-induced deletions

Our analysis examined the end product of mutagenesis. Therefore, the mutational patterns identified could reveal potential repair pathways involved when DNA is challenged with MMC-induced lesions. The data presented here indicate that MMC-DNA interactions resulting in deletions most likely involve interstrand crosslinks, as indicated by the bias toward 5′-CpG-3′ dinucleotides in the deleted regions of DNA (Figure 1). Previous studies have reported that the alkylation step of MMC, resulting in an interstrand crosslink, is absolutely specific for the duplex DNA sequence CpG•CpG (complementary CpG sequence on opposite strands of DNA) (Borowy-Borowski *et al.* 1990). It has been proposed that this specificity is due to the unique alignment of the monoalkylated guanine in the minor groove, and is not affected by the surrounding nucleotide context (Sastry *et al.* 1995; Gargiulo *et al.* 1995). Our analysis revealed MMC-induced deletions of various sizes, which may be due to nonhomologous end joining (NHEJ) (Simsek and Jasin 2010). NHEJ refers to the DNA repair process in which two double-strand break (DSB) ends are joined by ligation. The junctions of these DSBs are characterized by little or no microhomology, which has previously been suggested to help guide repair (Simsek and Jasin 2010). NHEJ includes both canonical NHEJ (C-NHEJ) and alternative non-homologous end joining (alt-NHEJ). However, the mechanisms differentiating these two processes in terms of mutagenesis are not entirely understood. Nevertheless, we identified deletions smaller than 5000 bp that were flanked by sequence microhomologies, which is more consistent with alt-NHEJ (Table 3). The molecular mechanisms of alt-NHEJ are not fully understood. However, it has been proposed that alt-NHEJ involves annealing microhomologies distant from the break site, resulting in deletions at the repair junctions (Guirouilh-Barbat *et al.* 2007). The deletions lacking microhomology (none or small) may have resulted from canonical NHEJ (C-NHEJ). While our data indicates the MMC-induced deletions are consistent with NHEJ in general, the line between alt-NHEJ and C-NHEJ is not entirely clear. Our results indicate the MMC-induced deletions in our strains may be a consequence of interstrand crosslink repair by way of NHEJ-mediated repair. The characterization of mutations identified in our study highlights the importance of sequence information in a biological system to understand the mechanism underlying mutations caused by commonly used chemotherapeutic agents such as MMC.
